# Exercise Improves Mitochondrial Homeostasis: A Potential Neuroprotective Strategy for Ischemic Stroke

**DOI:** 10.3390/antiox15050622

**Published:** 2026-05-14

**Authors:** Wenyan Bo, Qingxiang Guo, Wanyu Zhu, Yixuan Ma

**Affiliations:** 1 Institute of Physical Education, Shanxi University, Taiyuan 030006, China; 2 Institute of Sports and Exercise Biology, Shaanxi Normal University, Xi’an 710119, China

**Keywords:** mitochondrial homeostasis, ischemic stroke, exercise, oxidative stress

## Abstract

Regular exercise and physical activity are beneficial in reducing the risk and progression of ischemic stroke. However, the underlying physiological mechanisms by which exercise confers these protective effects remain incompletely understood. Disruption of mitochondrial homeostasis is key contributors to the pathophysiology of ischemic stroke. Exercise training effectively attenuates the onset and progression of ischemic stroke by significantly maintaining mitochondrial homeostasis, including improving mitochondrial biogenesis, balancing mitochondrial dynamics, maintaining mitochondrial redox, promoting mitophagy and mitochondrial transport. This review systematically summarizes the beneficial effects of exercise in the context of ischemic stroke and highlights the critical link between mitochondrial homeostasis disruption and stroke pathology. By providing a detailed analysis of the underlying molecular mechanisms, this study offers novel insights into exercise-based therapeutic strategies for ischemic stroke.

## 1. Introduction

Ischemic stroke (IS) is a common acute and life-threatening cerebrovascular disease characterized by high incidence and mortality, imposing a substantial burden on global healthcare systems and society [1]. Mitochondrial homeostasis disruption in the brain has been identified as an early and prominent feature of IS. Impairments in mitochondrial biogenesis, dynamics, oxidative stress regulation, and mitophagy further exacerbate neuronal injury and death [2,3]. Therefore, restoring mitochondrial homeostasis is considered a critical strategy for promoting neuronal functional recovery after IS. Exercise, as a safe and effective non-pharmacological intervention, has demonstrated significant neuroprotective potential in the treatment of IS. Accumulating evidence indicates that regular physical activity alleviates IS-related impairments by enhancing mitochondrial function [4,5,6]. However, the regulatory effects of exercise on mitochondrial function involve multiple signaling pathways and remain complex. Therefore, this review aims to systematically elucidate the molecular mechanisms by which exercise improves mitochondrial homeostasis in IS, with particular emphasis on mitochondrial biogenesis, dynamics, mitophagy, and oxidative stress. We hope to provide a solid theoretical foundation for developing personalized exercise strategies for the prevention and rehabilitation of IS.

## 2. Mitochondrial Homeostasis Imbalance After IS

### 2.1. Mitochondrial Biogenesis and Dynamics

**Figure 1:**
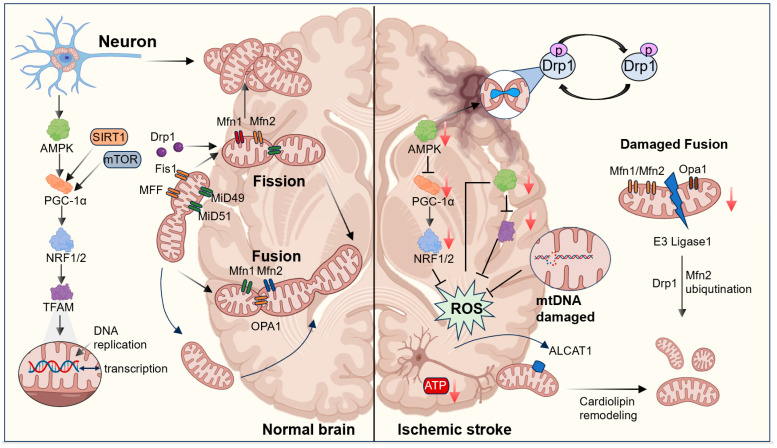
Mitochondrial dynamics and biogenesis in IS. Schematic comparison of mitochondrial regulation in normal and ischemic brain. Exercise activates AMPK–PGC-1α signaling to enhance mitochondrial biogenesis and maintain fission–fusion balance. In IS, excessive Drp1-mediated fission, impaired fusion, mtDNA damage, and ROS accumulation disrupt mitochondrial integrity.

Mitochondrial biogenesis is a fundamental process through which generate new mitochondria to replace damaged ones, such as those impaired by oxidative stress, thereby ensuring adequate energy supply (ATP) production and maintaining cellular energy homeostasis. It involves the coordinated formation of mitochondrial membranes, replication of mitochondrial DNA (mtDNA), and synchronized expression and assembly of proteins encoded by both nuclear and mitochondrial genomes, ultimately generating new functional mitochondria to meet cellular energy demands [7]. This process is tightly regulated by multiple signaling pathways, among which the AMP-activated protein kinase (AMPK)–peroxisome proliferator-activated receptor gamma coactivator-1α (PGC-1α) axis and the NAD^+^-dependent deacetylase sirtuin 1 (SIRT1)–PGC-1α pathway are considered central regulatory networks of mitochondrial biogenesis [7]. Under conditions of energy deficiency, AMPK is activated in response to an increased AMP/ATP ratio and enhances the transcriptional activity of PGC-1α through phosphorylation [8]. As a key transcriptional coactivator, PGC-1α further induces the activation of nuclear respiratory factors 1 and 2 (NRF 1 and NRF 2/GABP), which subsequently upregulate mitochondrial transcription factor A (TFAM), promoting mtDNA replication and transcription, thereby increasing mitochondrial content and enhancing oxidative phosphorylation capacity [9,10,11]. In addition, SIRT1 can deacetylate PGC-1α, further enhancing its transcriptional activity and facilitating mitochondrial biogenesis [12]. These signaling networks interact and counterbalance each other to finely regulate mitochondrial biogenesis (Figure 1).

However, following IS, cerebral ischemia and subsequent reperfusion injury severely disrupt this regulatory network. Ischemia-induced oxidative stress and mitochondrial dysfunction lead to mtDNA damage and reduced replication capacity, thereby suppressing mitochondrial biogenesis. Several studies have demonstrated that in experimental models of cerebral ischemia, the expression of key regulators such as PGC-1α, NRF1, and TFAM is significantly reduced, accompanied by decreased mtDNA copy number and impaired ATP production, ultimately exacerbating neuronal energy failure and cellular injury [10,13]. For instance, in the middle cerebral artery occlusion (MCAO) model, mtDNA copy number is markedly decreased, along with downregulation of TFAM, directly impairing mitochondrial transcription and biogenesis [14]. Conversely, activation of PGC-1α or enhancement of the AMPK–PGC-1α signaling axis has been shown to promote mitochondrial biogenesis, improve ATP production efficiency, and reduce infarct volume as well as neurological deficits [15,16]. Recent evidence further indicates that acyl-CoA: lysocardiolipin acyltransferase 1 (ALCAT1), under oxidative stress conditions, disrupts mitochondrial membrane stability by remodeling cardiolipin structure and aggravates mtDNA damage, whereas ALCAT1 deficiency increases mtDNA copy number and restores mitochondrial biogenesis capacity [17]. These findings suggest that ALCAT1 may represent a potential therapeutic target in IS.

Neurons exhibit a highly polarized morphology and possess substantial energy demands, requiring an intact and efficient mitochondrial network to sustain ATP production, calcium homeostasis, and apoptotic signaling. To accommodate these physiological requirements, mitochondria continuously undergo dynamic remodeling through cycles of fission and fusion, thereby maintaining mitochondrial quality control, optimizing metabolic efficiency, and adapting to local energy demands. Physiological levels of mitochondrial fission facilitate the segregation of damaged mitochondria and support mitochondrial quality control, thereby maintaining normal oxidative phosphorylation in neurons. However, under pathological conditions such as IS, mitochondrial fission is often excessively activated, resulting in mitochondrial fragmentation, loss of membrane potential, and reduced ATP production, which collectively exacerbate neuronal injury [18,19]. Supporting this view, inhibition of Drp1 has been shown to reduce infarct volume and improve neurological outcomes in stroke models [20,21]. The activity of Drp1 is regulated by multiple post-translational modifications, among which phosphorylation plays a critical role. Phosphorylation at Ser616 enhances Drp1 activity and promotes mitochondrial fission, whereas phosphorylation at Ser637 inhibits its activity [22]. In IS, Drp1 is frequently activated through dephosphorylation at Ser637, thereby promoting excessive mitochondrial fission [23]. In addition, A-kinase anchoring protein 1 (AKAP1), located on the outer mitochondrial membrane, recruits’ protein kinase A (PKA) to maintain phosphorylation of Drp1 at Ser637, thereby suppressing excessive fission, preserving mitochondrial respiratory chain function, reducing ROS production, and stabilizing calcium homeostasis. This mechanism has been recognized as neuroprotective in ischemic brain injury [24]. Recent studies have also shown that in microglia following stroke, alterations in mitochondrial fission factors such as MFF and Drp1 contribute to immune responses. T cell immunoglobulin and mucin domain-containing protein 4 (Tim4) can regulate microglial M1 polarization by modulating mitochondrial dynamics, thereby reducing infarct size and improving neurological function [25].

In contrast to mitochondrial fission, mitochondrial fusion is mediated by three major GTPases: mitofusin 1 (Mfn1), mitofusin 2 (Mfn2), and optic atrophy protein 1 (Opa1) [26]. Mfn1 and Mfn2 regulate outer mitochondrial membrane fusion, while Opa1 controls inner membrane fusion and maintains cristae structure integrity. Mfn1 and Mfn2 form transmembrane dimers or oligomers through their heptad repeat domains, facilitating the tethering and fusion of adjacent mitochondrial outer membranes [27]. Subsequently, Opa1 interacts with cardiolipin to mediate inner membrane fusion and preserve cristae architecture [28]. Recent evidence indicates that Opa1 levels decline with aging in both mice and humans and are further reduced following IS. Neuron-specific deletion of Opa1 exacerbates mitochondrial cristae disruption, increases infarct volume, and aggravates neuronal loss [29]. Moreover, mitochondrial E3 ubiquitin ligase 1 is upregulated after stroke and disrupts mitochondrial dynamics by promoting SUMOylation of Drp1 and ubiquitination of Mfn2, thereby aggravating brain injury [30]. In contrast, overexpression of Mfn2 significantly reduces hypoxia-induced apoptosis, while upregulation of Opa1 alleviates brain edema and neuronal injury following IS [31]. Restoring the balance between mitochondrial fission and fusion by downregulating Drp1 and Fis1 and upregulating Opa1, Mfn1, and Mfn2, thereby attenuating neuronal injury in cerebral ischemia–reperfusion injury (CIRI) [32]. Taken together, disruption of mitochondrial dynamics represents a critical mechanism underlying neuronal dysfunction after IS.

### 2.2. Mitochondrial Quality Control

**Figure 2:**
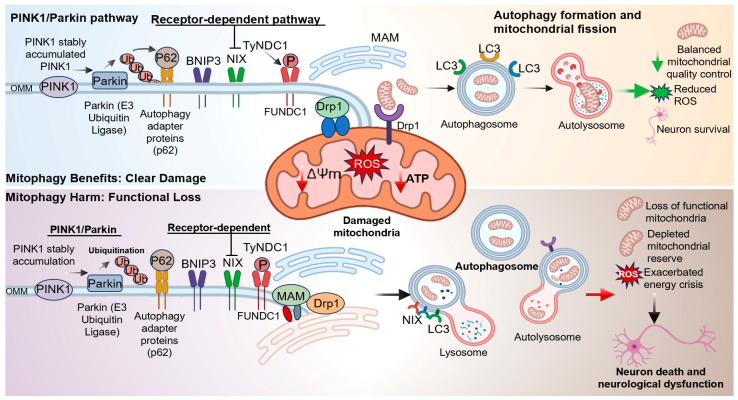
Dual role of mitophagy in ischemia/reperfusion-induced neuronal injury. Mitophagy is regulated by ubiquitin-dependent (PINK1/Parkin) and receptor-dependent pathways (BNIP3, NIX, FUNDC1), coordinating mitochondrial fission and autophagosome formation. Moderate activation promotes clearance of damaged mitochondria, reduces ROS, and preserves ATP production, thereby supporting neuronal survival. In contrast, excessive or dysregulated mitophagy results in loss of functional mitochondria, energy depletion, and aggravated neuronal injury.

Neuronal survival and functional stability critically depend on an intact mitochondrial quality control system. Among these processes, mitophagy defined as the selective removal of damaged, depolarized, or dysfunctional mitochondria, plays a pivotal role in maintaining mitochondrial homeostasis, limiting the accumulation of ROS, and preserving cellular energy metabolism [33]. Increasing evidence indicates that mitophagy exhibits distinct temporal dynamics and bidirectional effects at different stages of IS [34]. During the acute ischemic phase, the abrupt reduction in oxygen and substrate availability leads to impaired ATP production, loss of mitochondrial membrane potential, and suppression of mitochondrial biogenesis, ultimately resulting in severe metabolic disturbance [35]. In contrast, during reperfusion or recovery, excessive ROS generation and lipid peroxidation further aggravate mitochondrial damage. Under these conditions, moderate activation of mitophagy facilitates the timely clearance of damaged mitochondria, thereby alleviating oxidative stress and cellular injury [36]. However, excessive or sustained activation of mitophagy may result in the depletion of functional mitochondria, exhaustion of mitochondrial reserves, and exacerbation of energy crisis, ultimately promoting neuronal death and neurological dysfunction [37]. Therefore, mitochondrial quality control imbalance after stroke is not simply characterized by insufficient or excessive mitophagy, but rather reflects dysregulated mitophagy (Figure 2).

Mitophagy is generally categorized into ubiquitin-dependent and receptor-mediated pathways, among which the PTEN-induced kinase 1 (PINK1)/Parkin pathway and receptor-mediated mechanisms involving BNIP3, NIX (BNIP3L), and FUNDC1 are the most extensively studied [38,39]. Under physiological conditions, PINK1 is imported into mitochondria and degraded in the inner membrane. Upon mitochondrial damage induced by ischemia, hypoxia, or oxidative stress, loss of membrane potential prevents its import, leading to PINK1 accumulation on the outer mitochondrial membrane. This recruits the E3 ubiquitin ligase Parkin, promoting ubiquitination of outer membrane proteins and initiating mitophagy via autophagy adaptor proteins [40,41]. As a sensor of mitochondrial membrane potential (ΔΨm), PINK1 rapidly responds to ischemic stress and protects against mitochondrial dysfunction [42]. Consistently, Parkin deficiency exacerbates neuronal vulnerability and protein accumulation, whereas its upregulation enhances mitophagy, reduces brain injury, and improves neuronal survival after ischemia [43].

In addition, several studies have demonstrated that proteins such as PINK1, Beclin-1, and BNIP3 accumulate on the outer mitochondrial membrane (OMM) and mitochondria-associated membranes (MAMs) during mitophagy [44,45]. This accumulation promotes autophagosome formation and facilitates mitochondrial–endoplasmic reticulum interactions. Mitophagy is also largely mediated by the BNIP3/NIX pathway [46]. In mice, deletion of Bnip3L inhibits autophagy and exacerbates ischemia–reperfusion injury, whereas overexpression of BNIP3L reverses this effect [47]. Furthermore, the FUNDC1 pathway plays a critical role in coordinating mitochondrial fission and mitophagy. Prior to mitophagy, mitochondria typically undergo a certain degree of fragmentation, as smaller mitochondrial fragments are more readily engulfed by autophagosomes. Under hypoxic conditions, FUNDC1 functions as a key regulator at MAMs, coordinating mitochondrial fission and mitophagy [48,49]. Knockdown of FUNDC1 has been shown to increase mitochondrial number, induce elongation of mitochondrial morphology, and reduce the colocalization of mitochondria with autophagosomes, thereby significantly inhibiting mitophagy [50,51]. In the early stage of ischemia/reperfusion injury in neurons (within 3 h), FUNDC1 is activated through dephosphorylation at the Tyr18 site [52]. Under ischemic or hypoxic conditions followed by reperfusion, dephosphorylation of FUNDC1 at Tyr18 enhances its interaction with LC3 and promotes mitophagy [53,54]. In middle cerebral artery occlusion/reperfusion (MCAO/R) and oxygen–glucose deprivation/reperfusion (OGD/R) models, knockdown of FUNDC1 markedly suppresses mitophagy, increases infarct volume, and aggravates neurological deficits [55]. Tissue plasminogen activator (tPA), a major thrombolytic agent used clinically for IS, has been shown to improve mitochondrial function and reduce neuronal apoptosis via FUNDC1-mediated mitophagy [56]. Collectively, mitophagy exhibits a dual role following stroke, and its beneficial effects largely depend on maintaining an appropriate level of mitochondrial quality control.

### 2.3. Mitochondrial Oxidative Stress Imbalance

**Figure 3:**
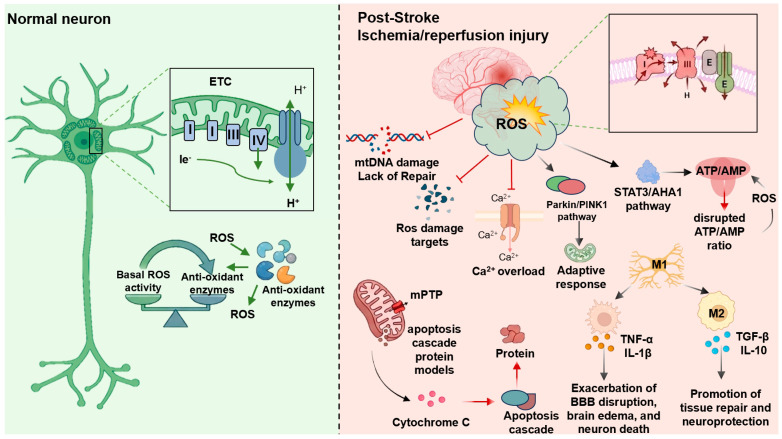
Mitochondrial oxidative stress imbalance after IS and neuronal damage Ischemia/reperfusion injury triggers excessive ROS production, primarily via damaged ETC complexes I–III, leading to a pathological cascade. Elevated ROS induces mtDNA damage, Ca^2+^ overload, and mPTP opening, which promotes cytochrome c release and apoptosis. Concurrently, ATP depletion and STAT3/AHA1 activation create a maladaptive feedback loop. As a second messenger, ROS modulates microglial polarization: the M1 phenotype exacerbates BBB disruption and neuronal death via TNF-α/IL-1β release, while PGC-1α/Piezo1 signaling promotes the M2 phenotype to secrete IL-10/TGF-β, facilitating neuroprotection and tissue repair.

Mitochondria are the primary source and regulatory hub of ROS in cells. Following stroke, the dynamic balance between ROS production and clearance is severely disrupted, making mitochondrial oxidative stress a key component of the ischemic cascade and a central contributor to neuronal injury [57] (Figure 3). During ischemia/reperfusion in the central nervous system, mitochondrial ETC complexes I and III are considered the major sources of ROS generation [58,59]. Excessive ROS can damage DNA, proteins, and lipids, disrupt Ca^2+^ homeostasis, induce mitochondrial depolarization, and ultimately trigger cell death pathways. In addition, ATP production is markedly reduced under ischemic conditions, further exacerbating energy failure and promoting neuronal death. Increasing evidence suggests that excessive mitochondrial oxidative stress is a critical determinant of neuronal death in the ischemic cascade [60]. In the chronic phase, although acute oxidative stress responses gradually subside, mitochondrial bioenergetic dysfunction persists, characterized by reduced electron transport efficiency, impaired mitochondrial dynamics, and disrupted coupling between energy supply and neuronal activity, thereby hindering functional recovery [61]. Similarly, in hemorrhagic stroke, elevated intracranial pressure induces mitochondrial oxidative stress, ATP depletion, and structural damage, while ROS also mediates endoplasmic reticulum stress, further aggravating mitochondrial dysfunction [62]. These persistent metabolic impairments contribute to long-term neurological deficits and limited recovery.

Beyond its damaging effects, ROS also functions as an important second messenger. ROS can activate the PINK1/Parkin pathway and induce mitophagy to eliminate severely damaged mitochondria, representing an adaptive response [63]. In MCAO models, deficiency of antioxidant enzyme peroxiredoxin 6 aggravates mitophagy after ischemic injury [64], whereas silencing of the ROS-regulated protein ShcA reduces mitophagy activation in photothrombotic stroke models [65]. In addition, mitophagy levels in neuronal mitochondria influence malondialdehyde (MDA) and SOD levels [66]. On the other hand, aberrant ROS signaling can trigger pathological responses. Ischemic stress activates the STAT3 pathway and upregulates AHA1 (activator of Hsp90 ATPase homolog 1). AHA1 translocates to mitochondria and interacts with ATP synthase, disrupting the ATP/AMP ratio and further promoting ROS production, thereby forming a vicious cycle that exacerbates brain injury. Silencing AHA1 can interrupt this cycle and improve outcomes [67]. In contrast, activation of PGC-1α promotes mitochondrial biogenesis and induces the expression of key antioxidant enzymes. Both in vitro and in vivo studies of cerebral ischemia/reperfusion injury (CIRI) demonstrate that PGC-1α activation reduces mitochondrial oxidative stress, maintains mitochondrial homeostasis, and protects neurons from ferroptosis-related damage [15].

Microglial inflammatory responses after stroke are also closely linked to mitochondrial oxidative stress. During IS, microglia are rapidly activated and participate in immune responses by releasing inflammatory mediators [68]. Microglia can polarize into pro-inflammatory M1 or anti-inflammatory M2 phenotypes. M1 microglia release pro-inflammatory cytokines such as TNF-α and IL-1β, whereas M2 microglia secrete anti-inflammatory factors such as IL-10 and TGF-β to promote tissue repair [69,70]. Excessive activation of M1 microglia contributes to sustained inflammation and aggravates brain injury, while M2 polarization alleviates inflammation and supports neuroprotection and repair [71]. Studies have shown that M1 microglial infiltration significantly increases in ischemic regions in MCAO models, whereas inhibiting M1 polarization or promoting M2 polarization alleviates brain injury and improves neurological function [25,72,73]. Microglia/macrophages and neurons play key roles in the production of pro-inflammatory cytokines during the inflammatory cascade [74], in which ROS acts as a critical regulator. Ischemia-induced ROS and inflammatory mediators activate and recruit microglia/macrophages in the ischemic core and penumbra. Activated microglia further release inflammatory factors, exacerbating blood–brain barrier disruption, cerebral edema, and neuronal death [75,76]. Inhibition of the ROS/NF-κB signaling axis has been shown to promote neuronal differentiation and improve functional recovery after IS [77]. Notably, the mechanosensitive protein Piezo1 in microglia can sense astrocyte stiffness and anisotropy after stroke, reduce glial scar rigidity, and promote neural regeneration, thereby improving motor recovery [78]. In addition, activation of Piezo1 enhances mitochondrial oxidative phosphorylation, respiration, and glycolysis, leading to increased ATP production [79], suggesting that Piezo1 may serve as a potential therapeutic target in stroke.

At the mitochondrial level, ischemia leads to a sharp reduction in oxygen supply, impairing electron acceptance by the ETC and severely limiting ATP production [80]. Meanwhile, excessive ROS generated by the ETC and NADPH oxidase (NOX) damages mtDNA, which is particularly vulnerable due to the lack of histone protection and limited repair capacity. ETC uncoupling further disrupts oxidative phosphorylation and worsens stroke outcomes [81]. In addition, oxidative stress induces cytosolic Ca^2+^ overload and promotes persistent opening of the mitochondrial permeability transition pore (mPTP), compromising mitochondrial membrane integrity and triggering the release of pro-apoptotic factors such as cytochrome c, ultimately leading to neuronal apoptosis [82,83,84]. Studies have shown that mitochondrial dysfunction, characterized by reduced membrane potential, ATP depletion, excessive ROS production, and mPTP opening, occurs in both OGD and dMCAO models, leading to increased neuronal apoptosis [19]. Emerging therapeutic strategies, such as intranasal mitochondrial transplantation, have been shown to improve mitochondrial function, inhibit apoptosis, and alleviate brain edema and blood–brain barrier disruption [85]. Furthermore, mitochondrial ETC activity of glycerol-3-phosphate dehydrogenase (G3PDH) is significantly reduced in peripheral blood of stroke patients, whereas functional electrical stimulation combined with exercise can reverse this impairment, restoring motor function and mitochondrial activity [86]. Therefore, suppressing excessive ROS production, enhancing antioxidant defenses, and restoring mitochondrial redox homeostasis may represent effective strategies for mitigating neuronal injury and promoting functional recovery after stroke.

### 2.4. Mitochondrial Transport Dysfunction

**Figure 4:**
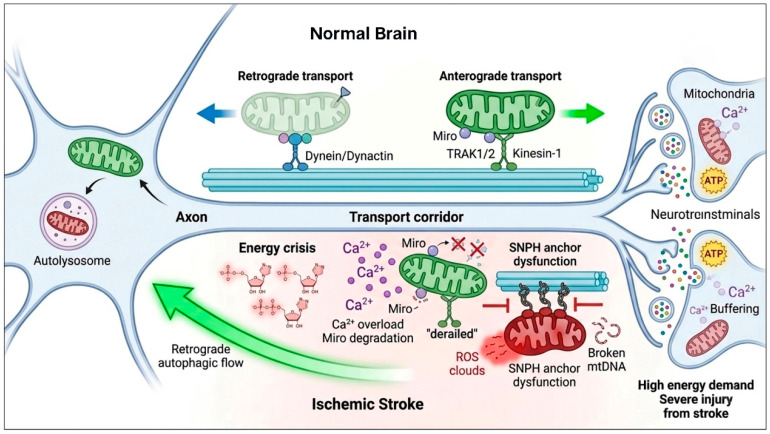
Disorder in axonal mitochondrial transport in IS. In the healthy state, mitochondria undergo long-distance transport along microtubule tracks; anterograde movement is driven by the Kinesin-1 motor complex via the Miro/TRAK adapter system, while retrograde transport is mediated by Dynein/Dynactin for somal degradation. Upon ischemic insult, the massive influx of Ca^2+^ triggers the proteolytic degradation of Miro, leading to the dissociation of Kinesin motors and subsequent mitochondrial “derailment.” Simultaneously, the upregulation of the docking protein Syntaphilin (SNPH) acts as a molecular “anchor” to immobilize damaged mitochondria, which are characterized by ATP depletion, excessive ROS production, and mtDNA fragmentation.

In neurons, mitochondria serve not only as the primary source of ATP but also play essential roles in metabolic processes, including the generation of tricarboxylic acid (TCA) cycle intermediates that act as precursors for neurotransmitters such as γ-aminobutyric acid (GABA) and glutamate [87]. In addition, mitochondria regulate synaptic vesicle release by buffering intracellular Ca^2+^ levels, thereby maintaining stable neurotransmission and neuronal network activity. Following IS, mitochondrial dysfunction and abnormal distribution disrupt these processes, leading to impaired synaptic transmission and neuronal dysfunction (Figure 4).

Mitochondrial distribution in neurons is regulated by a coordinated transport system responsive to local energy demands. Mitochondria are dynamically positioned in axons, dendrites, and synaptic terminals via microtubule-based transport. Anterograde transport is mediated by kinesin, while retrograde transport is driven by the dynein–dynactin complex. These motors are linked to mitochondria through TRAK1/2 and the outer membrane GTPase Miro, forming a transport complex that enables movement along microtubules [88]. This process is essential for ATP supply, synaptic function, Ca^2+^ homeostasis, and mitochondrial quality control [89]. Under physiological conditions, healthy mitochondria are recruited to regions with high energy demand, whereas damaged mitochondria are transported retrogradely to the soma for repair or degradation via mitophagy. However, in IS, this finely regulated transport system is severely disrupted. Following ischemia, ATP production in brain tissue declines rapidly [90], resulting in insufficient energy for synaptic processes such as vesicle cycling and ion pump activity required for membrane potential recovery. Consequently, synaptic function deteriorates rapidly, leading to neurological deficits.

Disruption of Ca^2+^ homeostasis is a key mechanism underlying mitochondrial transport impairment following IS. Under normal conditions, Miro proteins regulate mitochondrial motility by sensing intracellular Ca^2+^ levels [91]. However, during ischemia, intracellular Ca^2+^ levels rise sharply and persistently [92]. Excessive Ca^2+^ induces degradation of Miro, causing dissociation of mitochondria from motor protein complexes and leading to abnormal mitochondrial arrest at sites of injury, thereby impairing their transport capacity [91]. These dysfunctional mitochondria release excessive ROS and mtDNA, further promoting neuronal apoptosis. In addition, Ca^2+^ overload activates calpain, which cleaves motor and adaptor proteins, further disrupting the transport machinery.

Interestingly, retrograde mitochondrial transport may exert a protective effect after IS. Enhanced retrograde transport has been shown to alleviate axonal injury in oxygen–glucose deprivation (OGD) models, likely by facilitating the clearance of damaged mitochondria via mitophagy [93]. In contrast, excessive mitochondrial anchoring impairs this process. For instance, the anchoring protein syntaphilin (SNPH) immobilizes mitochondria on axonal microtubules; its overexpression inhibits retrograde transport and mitophagy, thereby exacerbating mitochondrial dysfunction and neuronal injury [94]. These findings suggest that increased retrograde transport may represent a compensatory response to ischemic stress. Nevertheless, the regulatory mechanisms and therapeutic targets of mitochondrial transport in IS remain incompletely understood and warrant further investigation.

## 3. Molecular Mechanisms by Which Exercise Promotes Recovery from IS Through Regulation of Mitochondrial Homeostasis

Exercise is widely recognized as an effective intervention for improving prognosis and promoting functional recovery after stroke. Regular physical activity not only reduces the risk of cardiovascular diseases but also plays a critical role in both the prevention and rehabilitation of stroke [95,96,97]. Follow-up data from the National Health and Nutrition Examination Survey (NHANES) indicate that physical inactivity is significantly associated with an increased risk of stroke [98]. Moreover, a large prospective cohort study involving approximately one million women in the United Kingdom demonstrated that regular moderate-intensity physical activity reduces the risk of coronary heart disease and cerebrovascular diseases [99]. Willey et al. further reported that moderate-to-vigorous physical activity is associated with a reduced risk of stroke in men, whereas the association in women remains less clear [100]. Currently, sex differences in the preventive effects of exercise on stroke remain controversial, and the underlying mechanisms require further investigation [101]. In addition, studies in hypertensive populations have shown that participation in moderate-to-vigorous physical activity significantly reduces the incidence of stroke [102]. In the context of clinical rehabilitation, exercise training has been shown to markedly improve motor function, cognitive performance, emotional status, and quality of life in stroke survivors [103,104], highlighting its substantial clinical value.

**Figure 5:**
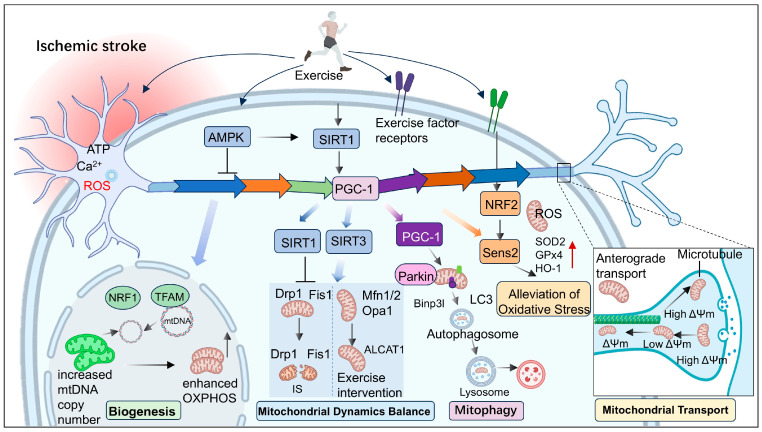
Exercise-mediated regulation of mitochondrial homeostasis in IS. Schematic illustration of the mechanisms by which exercise confers neuroprotection through modulation of mitochondrial homeostasis following IS. Exercise-induced factors (exerkines) activate key signaling pathways to enhance mitochondrial biogenesis, antioxidant defense, and oxidative phosphorylation. Concurrently, exercise restores mitochondrial dynamics by balancing fission and fusion processes, and promotes mitophagy to facilitate the clearance of damaged mitochondria. In addition, exercise improves mitochondrial transport by maintaining membrane potential and reducing Ca^2+^ overload, thereby ensuring adequate energy supply at synaptic sites. Collectively, these coordinated contribute to improved neuronal survival and functional recovery after IS.

In addition to clinical evidence, preclinical studies have further revealed the potential neuroprotective mechanisms of exercise in IS. Multiple studies have demonstrated that exercise interventions, including treadmill training, voluntary wheel running, and exercise preconditioning, significantly reduce infarct volume and improve neurological outcomes [105,106,107,108,109]. These beneficial effects are associated with suppression of neuroinflammation, attenuation of oxidative stress, modulation of autophagy, and reduction in neuronal apoptosis [106,110,111]. Notably, the neuroprotective effects of exercise are largely mediated by the regulation of mitochondrial homeostasis. Exercise promotes mitochondrial biogenesis, regulates mitochondrial dynamics, alleviates oxidative stress, and enhances mitochondrial quality control, thereby reducing neuronal injury and facilitating functional recovery after cerebral ischemia (Table 1; Figure 5). Although these findings still lack sufficient clinical evidence, they suggest that targeting mitochondrial function through exercise-based interventions may represent a promising strategy for the prevention and treatment of IS.

Although these preclinical findings support the beneficial effects of exercise in alleviating IS injury through the regulation of mitochondrial homeostasis, different IS models may exhibit distinct pathological characteristics and differential responses to exercise interventions. The tMCAO model more closely resembles clinical ischemia–reperfusion injury and is characterized by pronounced oxidative stress, calcium overload, and excessive mitochondrial ROS production. Therefore, exercise interventions in tMCAO models are often associated with enhanced mitochondrial antioxidant capacity and improved mitochondrial biogenesis. In contrast, the permanent MCAO model lacks reperfusion and is characterized by sustained ischemia, severe ATP depletion, and progressive neuronal necrosis. Under these conditions, the neuroprotective effects of exercise may be more closely related to the promotion of angiogenesis and enhancement of long-term mitochondrial quality control.

In addition, exercise timing, intensity, and duration may produce different outcomes across these models. In tMCAO models, moderate exercise initiated during an appropriate recovery window may better facilitate mitochondrial remodeling and neurological recovery, whereas early high-intensity exercise after permanent MCAO may exacerbate metabolic stress and neuronal injury. Regarding the preventive effects of exercise, most studies suggest that approximately 14–21 days of exercise training is sufficient to induce ischemic preconditioning. In terms of rehabilitative effects, as little as 5 days of exercise may promote mitochondrial biogenesis in ischemic neurons; however, sustained neuroprotective effects may require longer intervention periods. Furthermore, gender differences are also an important cause of the diverse outcomes. Currently, most preclinical studies mainly use young male animals, while studies on female animals are relatively scarce. Estrogen plays a significant role in regulating mitochondrial metabolism, oxidative stress responses, and neuronal survival. Therefore, female animals may differ from male animals in terms of mitochondrial adaptability and motor responsiveness, which could affect the neuroprotective effect mediated by exercise. These differences may partly explain the inconsistency observed among experimental studies investigating exercise-mediated mitochondrial regulation before and after IS.

### 3.1. Exercise Promotes Mitochondrial Biogenesis

Mitochondrial biogenesis is a fundamental process for maintaining mitochondrial quantity and function. Exercise is widely recognized as an important physiological stimulus that promotes mitochondrial biogenesis. During exercise, metabolic stress, mechanical stimulation, and local hypoxia activate multiple intracellular signaling pathways, thereby inducing mitochondrial generation and improving cellular energy metabolism [119,120]. Among the molecular regulators of mitochondrial biogenesis, peroxisome proliferator-activated receptor gamma coactivator-1α (PGC-1α) is considered a central mediator. As a key transcriptional coactivator, PGC-1α promotes mitochondrial biogenesis and enhances oxidative phosphorylation by regulating the expression of mitochondrial-related genes and mtDNA copy number [121]. Studies have shown that endurance exercise significantly upregulates PGC-1α mRNA and protein expression in skeletal muscle, thereby promoting mitochondrial biogenesis [122]. In addition, SIRT1, a key member of the mitochondrial deacetylase family, plays a crucial role in regulating mitochondrial function and energy metabolism [123]. SIRT1 enhances the transcriptional activity of PGC-1α through deacetylation, forming the SIRT1–PGC-1α signaling axis. This axis is essential for maintaining mitochondrial homeostasis, regulating energy metabolism, and resisting oxidative stress, and its dysfunction has been closely associated with various pathological conditions, including cardiovascular diseases, diabetes, and neurodegenerative disorders [124]. Therefore, activation of this pathway represents an important mechanism underlying the beneficial effects of exercise. Indeed, exercise has been shown to effectively activate the SIRT1/PGC-1α signaling pathway, thereby enhancing mitochondrial biogenesis and improving mitochondrial function [125]. Meanwhile, AMPK a key sensor and regulator of cellular energy metabolism, is rapidly activated in response to exercise and participates in metabolic reprogramming and mitochondrial regulation [126,127]. On the one hand, AMPK directly promotes mitochondrial biogenesis through phosphorylation of PGC-1α [127]; on the other hand, in cerebral ischemic injury, AMPK exerts neuroprotective effects by modulating immune cell activity and inducing protective autophagy [128,129]. Notably, AMPK and SIRT1 exhibit significant functional synergy. AMPK enhances SIRT1 activity by increasing intracellular NAD^+^ levels, while SIRT1 further promotes PGC-1α transcriptional activity. Together, they form the AMPK–SIRT1–PGC-1α regulatory axis, which plays a critical role in maintaining mitochondrial homeostasis under conditions of energy stress [124,130]. Experimental studies further support these molecular mechanisms. In a photothrombotic stroke model, intranasal mitochondrial transplantation activates AMPK and the SIRT1/PGC-1α signaling pathway, thereby exerting neuroprotective effects by reducing oxidative stress and inflammation [131]. In a MCAO model, four weeks of treadmill training activates the SIRT1/BDNF/mTORC1 signaling pathway, significantly attenuating neuroinflammation and improving post-stroke depressive-like behaviors [132]. Similarly, Zhao et al. (2023) demonstrated that exercise preconditioning improves outcomes in MCAO mice by activating AMPK–mTOR and AMPK–FOXO3a–SKP2–CARM1 signaling pathways, enhancing autophagic flux, and suppressing neuroinflammation and oxidative stress [105]. In addition, mitochondrial TFAM also plays a key role in mitochondrial biogenesis [133,134]. Studies have shown that TFAM expression is significantly reduced following cerebral ischemia–reperfusion injury, whereas two weeks of enriched environment training markedly increases TFAM levels, promotes mitochondrial biogenesis in the brain, and alleviates neuronal dysfunction [135]. Importantly, exercise during the recovery phase of stroke has been shown to upregulate TFAM expression. For instance, exercise intervention as early as 7 days after stroke increases the expression of TFAM, NRF-1, and COXIV in the brain, thereby enhancing mitochondrial biogenesis and improving neurological function [113]. In summary, exercise training enhances mitochondrial biogenesis through coordinated activation of multiple signaling pathways, particularly the AMPK–SIRT1–PGC-1α axis, along with upregulation of key regulators such as TFAM, ultimately improving mitochondrial function and restoring mitochondrial homeostasis.

### 3.2. Exercise Improves Mitochondrial Dynamics

In neurological injuries such as IS, mitochondrial dynamics are often characterized by excessive fission and insufficient fusion, leading to mitochondrial fragmentation, increased oxidative stress, and neuronal death. Therefore, restoring the balance of mitochondrial dynamics is considered an important strategy for alleviating post-stroke neuronal injury. Recent studies have demonstrated that exercise training can significantly regulate the expression of proteins involved in mitochondrial dynamics, thereby restoring mitochondrial network integrity and improving cellular function [136]. 12 weeks of endurance running or voluntary exercise markedly increased the protein levels of Mfn1 and Mfn2 in multiple brain regions, while reducing Drp1 expression [137]. Similarly. Koo et al. [138] found that high-fat diet-induced obesity disrupted mitochondrial dynamics in rat hippocampal neurons, whereas 8 weeks of treadmill exercise effectively reversed this imbalance, as evidenced by decreased expression of fission-related proteins Drp1 and Fis1, and increased levels of fusion proteins Mfn1, Mfn2, and Opa1, accompanied by significant improvements in mitochondrial function. The regulatory effects of exercise on mitochondrial dynamics are not limited to changes in protein expression but also involve post-translational modifications of key regulatory proteins. Exercise can modulate the phosphorylation status of Drp1 through activation of the AMPK/SIRT1 signaling pathway, thereby inhibiting excessive mitochondrial fission and maintaining mitochondrial network stability [117]. These mechanisms are critical for the restoration of mitochondrial function following ischemic injury. Gusdon and colleagues found that a 3-week exercise intervention did not induce significant increases in mitochondrial protein expression or mitochondrial biogenesis in the brains of either young or aged mice [139]. Likewise, Kitaoka et al. observed no obvious alterations in mitochondrial dynamics-related proteins following acute resistance exercise in rats [140]. In contrast, prolonged resistance training for 4 weeks markedly elevated the expression levels of Mfn1, Mfn2, and Opa1 in the gastrocnemius muscle. Consistently, a 10-week resistance training program also enhanced the expression of these mitochondrial fusion proteins in the skeletal muscle of older adults [141]. Collectively, these findings indicate that exercise-induced modulation of mitochondrial dynamics may depend on multiple factors, including exercise intensity, duration, and modality, and may additionally be influenced by age, tissue specificity, pathological conditions such as obesity, and differences in experimental settings.

In stroke models, the protective effects of exercise preconditioning on mitochondrial dynamics are particularly evident. Moderate enhancement of mitochondrial fusion and suppression of excessive fission contribute to reduced oxidative stress, inflammation, and neuronal apoptosis, ultimately alleviating ischemic brain injury. Two weeks of exercise preconditioning in rats increased Opa1 expression in the brain and attenuated post-ischemic cerebral edema [114]. Consistently, exercise-induced upregulation of Opa1 has been shown to reduce brain edema following IS [31]. Qin et al. [117] further demonstrated in an aged female mouse model of cerebral ischemia/reperfusion (I/R) that 9 weeks of voluntary wheel running preconditioning significantly regulated the expression of Mfn2 and Drp1, resulting in reduced infarct volume and improved neurological function. Collectively, these findings indicate that exercise exerts neuroprotective effects in stroke by restoring mitochondrial dynamic balance.

### 3.3. Exercise Modulates Mitophagy in IS

Accumulating evidence indicates that exercise not only promotes mitochondrial biogenesis but also facilitates mitochondrial renewal by regulating mitophagy, thereby maintaining the dynamic balance of mitochondrial quality control. Notably, even short-term acute exercise has been shown to enhance mitophagic activity. For instance, a previous study demonstrated that approximately 90 min of acute exercise significantly increased mitophagic flux and promoted the selective clearance of damaged mitochondria, ultimately supporting mitochondrial turnover and functional integrity [142]. In addition, exercise-induced PGC-1α, a key regulator of mitochondrial biogenesis, also plays a crucial role in mitophagy regulation. Evidence suggests that resistance exercise activates the hippocampal PGC-1α/BDNF/Akt/glycogen synthase kinase-3β (GSK-3β) signaling pathway, thereby enhancing mitophagy, promoting mitochondrial biogenesis, and improving synaptic plasticity in aged mice [143]. PINK1 is a pivotal regulator of Parkin-dependent mitophagy and represents a major focus in IS research [39]. Upon mitochondrial damage, PINK1 accumulates on the outer mitochondrial membrane, leading to the phosphorylation and activation of Parkin. Activated Parkin subsequently interacts with LC3 to initiate mitophagy. It has been reported that exercise upregulates SIRT1 expression and activates the PINK1/Parkin signaling pathway, thereby enhancing hippocampal mitophagy and alleviating depressive-like behaviors induced by chronic social defeat stress (CSDS) [144]. Furthermore, Sirtuin family member SIRT3 also plays a critical role in exercise-mediated regulation of mitophagy. Studies have shown that exercise markedly increases SIRT3 expression in skeletal muscle, which in turn activates the PINK1/Parkin pathway, enhances mitophagic flux, and promotes ATP production as well as mitochondrial metabolic enzyme activity [106]. Importantly, the role of mitophagy may vary across different stages following IS. Nevertheless, exercise preconditioning has been shown to upregulate mitophagy-related proteins, including Bnip3L and Parkin, in the ischemic cortex of female mice, leading to significant improvements in neurological deficits in ischemia/reperfusion (I/R) models [117].

### 3.4. Exercise Alleviates Mitochondrial Oxidative Stress in IS

Mitochondrial oxidative stress is a critical component of the pathophysiology of IS, primarily characterized by excessive production of ROS and impaired antioxidant defense systems. A substantial body of evidence demonstrates that exercise can markedly reduce oxidative stress following IS by enhancing antioxidant capacity and improving mitochondrial function. Regular exercise directly strengthens endogenous antioxidant defenses. Studies have shown that exercise upregulates both the expression and activity of key antioxidant enzymes, including superoxide dismutase 2 (SOD2) and glutathione peroxidase (GPx), thereby facilitating efficient ROS scavenging [145,146]. Meng et al. (2024) reported that exercise preconditioning significantly attenuated oxidative damage in the cerebral cortex of MCAO rats, as evidenced by reduced MDA levels and increased activities of glutathione peroxidase (GSH-Px) and SOD [147]. The regulatory effects of exercise on mitochondrial oxidative stress have also been validated in clinical studies. Stroke patients undergoing four weeks of exercise training exhibited significant improvements in mitochondrial ETC function and OXPHOS capacity in platelets, accompanied by a marked reduction in mitochondrial ROS production. These changes collectively contributed to decreased systemic oxidative stress and inflammatory responses [148]. At the molecular level, exercise mitigates oxidative stress partly through the induction of various exercise-induced factors (exerkines). For example, in MCAO models, exercise has been shown to upregulate SIRT1 expression and modulate ROS/ER stress signaling pathways, thereby exerting neuroprotective effects [149]. In addition, the myokine irisin, released during exercise, can activate NRF2 and its downstream targets NAD(P)H quinone dehydrogenase 1 (NQO-1) and heme oxygenase-1 (HO-1), leading to reduced mitochondrial damage, inflammation, and oxidative stress, as demonstrated in traumatic brain injury models [150]. Exercise interventions may also exert neuroprotective effects by upregulating Sestrin2 (Sesn2), thereby inhibiting oxidative stress and neuroinflammation [106]. Notably, recent studies have identified ALCAT1 as an important regulator of mitochondrial oxidative stress. Overexpression of ALCAT1 disrupts mitochondrial respiratory chain supercomplex assembly and increases ROS production [149]. Conversely, pharmacological inhibition of ALCAT1 (e.g., with Dafa) suppresses ROS generation, enhances mitochondrial respiration, and improves cardiac function in ischemic models [151]. Although direct evidence linking exercise to ALCAT1 regulation in IS brain tissue remains limited, this molecule may represent a promising therapeutic target for future investigations.

### 3.5. Exercise Participates in Mitochondrial Transport

Mitochondria are highly dynamic organelles that undergo continuous movement within cells. In neurons, mitochondria generated in the soma are transported along axons and dendrites to provide ATP required for synaptic activities, including synaptogenesis and neurotransmitter release [152]. During axonal transport, mitochondria undergo constant fission, fusion, and morphological remodeling, which facilitate their distribution among the soma, axons, dendrites, and synaptic terminals [153]. During IS, rapid ATP depletion severely impairs mitochondrial transport systems, leading to insufficient mitochondrial supply at synaptic sites and exacerbating neuronal injury. Emerging evidence suggests that abnormalities in mitochondrial dynamics are a key contributor to impaired mitochondrial transport. Excessive activation of Drp1 induces mitochondrial fragmentation and disrupts mitochondrial network integrity, thereby hindering axonal transport [154,155,156]. Exercise training has been proposed to improve mitochondrial transport by modulating mitochondrial dynamics. A three-week exercise intervention significantly increased Drp1 protein levels in the brains of aged mice, suggesting enhanced mitochondrial transport capacity [139]. Furthermore, mitochondrial transport exhibits a bidirectional nature. Typically, mitochondria with high membrane potential (ΔΨm) preferentially undergo anterograde transport toward synapses, whereas damaged mitochondria with reduced membrane potential are transported retrogradely to the soma for degradation [157]. Exercise has also been shown to suppress Ca^2+^ overload in neurons following ischemia, thereby preserving mitochondrial structure and maintaining transport capacity [5]. Similarly, exercise preconditioning enhances mitochondrial respiratory function and alleviates I/R injury [118]. These adaptations collectively facilitate mitochondrial trafficking and reduce the accumulation of dysfunctional mitochondria within axons.

Of note, growth differentiation factor-5 (GDF-5) has recently been implicated in the regulation of mitochondrial function. Studies indicate that GDF-5 upregulates nucleoside diphosphate kinase A (NME1) expression and enhances mitochondrial respiration, thereby promoting neurite outgrowth [158]. Moreover, GDF-5 exhibits neuroprotective effects in various neurological disorders [159], and its upregulation has been shown to improve cardiac function in ischemic heart models [160]. It is therefore plausible that GDF-5 plays a role in the pathological progression of IS. Interestingly, our previous (unpublished) findings suggest that exercise modulates cortical GDF-5 expression. Accordingly, GDF-5 may represent a potential mediator of exercise-induced neuroprotection in IS, although its precise mechanisms warrant further investigation.

### 3.6. Exercise Regulates Mitochondrial Homeostasis in Non-Neuronal Cells After IS

Although neurons are considered the primary targets of ischemic injury, the brain is composed of multiple cell types, including glial cells (astrocytes and oligodendrocytes), microglia, endothelial cells, and pericytes. These cell populations also play critical roles in the pathophysiology and recovery processes of IS [5,161,162,163]. Following cerebral ischemia, astrocytes undergo reactive transformation and participate in metabolic support, neurovascular regulation, and antioxidant defense [164]. Aerobic exercise has been shown to enhance mitochondrial quality in astrocytes and promote the transfer of healthy mitochondria from astrocytes to neurons [165], thereby contributing to neuronal protection and maintenance of energy metabolism. In addition, microglial phenotypes dynamically change after IS, with M1-type (pro-inflammatory) microglia gradually increasing within 14 days, whereas M2-type (anti-inflammatory) microglia initially increase and subsequently decline [166]. Notably, the polarization state of microglia is closely associated with mitochondrial metabolism [167]. Exercise can suppress excessive activation of M1-type pro-inflammatory microglia/macrophages while promoting M2-type anti-inflammatory microglia [168], which may be partly related to the regulation of mitochondrial metabolism and reactive oxygen species production [169,170].

Furthermore, endothelial cells and pericytes are essential components of the neurovascular unit and are closely associated with BBB integrity and post-stroke angiogenesis [161,171]. Endothelial cells exhibit relatively strong resistance to ischemic injury, and a greater number of endothelial cells survive in the infarct region following early reperfusion [172]. IS induces the death of brain endothelial cells (BECs) and disrupts the integrity of BBB tight junctions. Notably, BECs contain approximately five times more mitochondria than peripheral endothelial cells [173], and this elevated mitochondrial burden is essential for maintaining cellular homeostasis and BBB integrity [174]. In addition, mitochondrial dysfunction and activation of apoptosis/necrosis pathways in brain microvascular endothelial cells (BMECs) directly contribute to BBB disruption [175]. Exercise has been reported to improve vascular function in hypertension by restoring endothelial mitochondrial dynamics and suppressing mitochondrial fragmentation [176]. Therefore, exercise may improve vascular remodeling and tissue repair after IS through the regulation of mitochondrial homeostasis in these vascular-associated cells.

Meanwhile, regionally activated stem cells, such as injury-induced neural stem/progenitor cells (iNSPCs) and injury-induced stem cells (iSCs), emerge within ischemic regions after stroke [177]. iNSPCs and iSCs are capable of differentiating into electrophysiologically functional neurons and contribute to neural repair following brain injury [177,178]. However, evidence regarding mitochondrial alterations in these cells after IS remains limited. Nevertheless, it is worth noting that stem cell activation and differentiation are highly energy-dependent processes. Therefore, mitochondrial homeostasis may also represent an important target through which exercise promotes regenerative responses after IS.

## 4. Conclusions and Prospects

Although preclinical studies have demonstrated that exercise can promote functional recovery of ischemic neurons by restoring mitochondrial homeostasis, including the regulation of mitochondrial biogenesis, mitochondrial dynamics, mitophagy, and oxidative stress, several limitations still exist regarding its clinical translation. First, animal studies are typically conducted using relatively young and healthy animals under highly controlled experimental conditions, whereas patients with IS are often older and commonly present with comorbidities such as hypertension, cardiovascular disease, and diabetes. These comorbid factors may significantly influence mitochondrial function, exercise tolerance, and rehabilitation outcomes. In addition, exercise interventions in animal studies are generally standardized and strictly supervised, whereas exercise adherence, rehabilitation accessibility, and individual tolerance vary considerably among clinical patients. Importantly, it remains unclear what degree of exercise intervention, including exercise intensity, duration, frequency, and modality, is sufficient to effectively activate mitochondrial function after IS. Moreover, the threshold intensity, duration, and frequency required to achieve maximal mitochondrial benefits may differ depending on disease stage, age, comorbidities, and functional status. Furthermore, patients frequently experience sequelae after IS, such as paresis, hemiplegia, and motor dysfunction, which may limit their ability to perform and/or tolerate conventional exercise interventions. Severe motor impairment may particularly restrict the feasibility of moderate- to high-intensity exercise protocols during the acute and early subacute phases after stroke. Therefore, additional large-scale clinical studies are needed to validate the translational applicability of exercise-mediated mitochondrial regulation in IS. Future studies should focus on developing safe and effective individualized exercise prescriptions based on patients’ neurological condition, cardiopulmonary function, rehabilitation stage, and overall exercise tolerance. Moreover, alternative rehabilitation strategies suitable for severely disabled patients should also be explored, including neuromuscular electrical stimulation, passive exercise, blood flow restriction training, robot-assisted rehabilitation, and low-intensity interval exercise protocols.

Notably, the current evaluation of post-stroke exercise interventions mainly relies on neurological functional scales, such as the NIH Stroke Scale (NIHSS) and modified Rankin Scale (mRS), as well as imaging-based assessments. However, these evaluation systems are often insufficient to sensitively and dynamically capture metabolic alterations at the cellular and subcellular levels. Therefore, the development of translatable biomarkers capable of dynamically reflecting mitochondrial functional status and exercise responsiveness is of great significance for advancing precision rehabilitation. First, platelet mitochondrial bioenergetic parameters have emerged as one of the most promising candidates for clinical translation. Platelets contain functionally intact mitochondria, and their metabolic characteristics may partially reflect systemic mitochondrial function. Previous studies have demonstrated that patients in the acute phase of IS often exhibit impaired oxidative phosphorylation capacity, reduced ATP production, and dysfunction of respiratory chain complexes. Regular exercise training may partially restore this metabolic phenotype by improving electron transport efficiency, increasing maximal respiratory capacity, and enhancing mitochondrial reserve capacity [148]. High-resolution respirometry (e.g., Oroboros or Seahorse systems) may enable early detection of metabolic responses to exercise. Future longitudinal and randomized studies are needed to validate their associations with neurological recovery and prognosis. Although not a direct surrogate for brain mitochondria, platelet-based measures provide a practical and repeatable peripheral metabolic window.

In addition, circulating extracellular vesicles (EVs) and mitochondria-related particles may serve as critical mediators linking exercise signals to metabolic adaptations in brain tissue. Recent studies have shown that platelet-rich plasma-derived EVs from exercise-trained donors can improve neuronal survival and calcium homeostasis under oxygen–glucose deprivation conditions in vitro, and promote functional recovery, reduce infarct size, and attenuate neuronal apoptosis in animal models of chronic IS [179]. These findings suggest that EVs may act as key intermediaries of exercise signaling and represent potential therapeutic alternatives when physical activity is limited [180]. However, challenges such as source heterogeneity and lack of standardized detection methods remain, necessitating further studies to define EVs subpopulations and establish unified analytical protocols.

Furthermore, exercise-induced circulating factors (exerkines) are emerging as key mediators of precision exercise prescriptions. Several exerkines, including FGF-21, irisin, BDNF, and lactate. However, their dynamic profiles and dose–response relationships in stroke remain unclear. Future studies should integrate multi-exerkine monitoring and multivariate analyses to clarify their associations with neurological recovery and mitochondrial metabolism, thereby enabling the development of individualized, mechanism-based rehabilitation strategies. Future studies integrating multi-biomarker profiling and longitudinal analyses are needed to clarify these dose–response relationships and optimize individualized exercise prescriptions, ultimately advancing mechanism-based and precision rehabilitation strategies for IS.

## Figures and Tables

**Table 1:** Impact of exercise on mitochondrial homeostasis and stroke outcomes in preclinical models.

Research	Stroke Model	Exercise Method	Mechanistic Pathways and Outcome
Zhang et al. (2012) [112]	Rats, tMCAO for 60 min.	Post-MCAO, treadmill running, 12 m/min, 30 min/day, 5 days	↑PGC-1 and NRF-1 mRNA ↑Mitochondrial biogenesis
Zhang et al. (2012) [113]	Rats, tMCAO for 90 min.	Post-MCAO, treadmill running, 12 m/min, 30 min/day, 7 days	↑PGC-1, mtDNA ↑Mitochondrial biogenesis
Zhang et al. (2014) [114]	Rats, tMCAO for 90 min.	Post-MCAO, treadmill running, 20 m/min, lasting 30 min/day for 2 weeks	↑OPA1, Mitochondrial dynamics ↓Brain edema
Li et al. (2019) [115]	Rats, tMCAO for 60 min.	Post-MCAO, voluntary exercise, 31 days	↑Mitochondrial function ↑Neurological function
Pan et al. (2021) [116]	Rats, tMCAO for 120 min	Post-MCAO, treadmill running, 0° slope, 10 m/min, 30 min/day, 7 days,	↓Neurobehavioral score ↓Cerebral infarction volume ↓Mitochondrial release of Cyt-C ↑Caveolin-1
Qin et al. (2023) [117]	Mouse, tMCAO for 60 min	Pre-MCAO, voluntary exercise, running speed was 12/15 rpm, 30 min/ day, 5 day/week, 9 weeks	↑Bnip3L, Parkin, LC3-II/LC3-I, LC3-II, p62, Atg7, Mfn2, Drp1 ↑Mitochondrial dynamics
Liang et al. (2024) [118]	Rat, MCAO/R for 60 min	Pre-MCAO, treadmill running, 15 m/min, 30 min/day, 5 day/week, 3 weeks	↑COX4, NAMPT, AMPK ↑OXPHOS,PPP ↑Mitochondrial respiration
Wu et al. (2024) [111]	Rat, MCAO/R for 60 min	Pre-MCAO, treadmill running, 20 m/min, 30 min/day, 5 day/week, 3 weeks	↑AMPK/PGC1α/GLUT4 ↑Mitochondrial morphology and function ↓Neuronal apoptosis
Li et al. (2025) [6]	Mouse, tMCAO for 60 min	Post-MCAO, voluntary exercise, 7 days	↑OPA1, Drp1, FIS1 ↑Mitochondrial dynamics ↓Mitochondrial apoptotic pathway ↑Neurological function

↑ indicates increase, and ↓ indicates decrease.

## Data Availability

No new data were created or analyzed in this study. Data sharing is not applicable to this article.
